# Recent Application of Zebrafish Models in Atherosclerosis Research

**DOI:** 10.3389/fcell.2021.643697

**Published:** 2021-02-25

**Authors:** Dandan Tang, Fang Geng, Chunxiao Yu, Ruilin Zhang

**Affiliations:** ^1^School of Basic Medical Sciences, Wuhan University, Wuhan, China; ^2^School of Life Sciences, Fudan University, Shanghai, China

**Keywords:** zebrafish model, atherosclerosis, dynamic monitoring, drug screening, risk factor assessment

## Abstract

Atherosclerotic cardiovascular disease is one of the leading causes of death worldwide. Establishing animal models of atherosclerosis is of great benefit for studying its complicated pathogenesis and screening and evaluating related drugs. Although researchers have generated a variety of models for atherosclerosis study in rabbits, mice and rats, the limitations of these models make it difficult to monitor the development of atherosclerosis, and these models are unsuitable for large scale screening of potential therapeutic targets. On the contrast, zebrafish can fulfill these purposes thanks to their fecundity, rapid development *ex utero*, embryonic transparency, and conserved lipid metabolism process. Thus, zebrafish have become a popular alternative animal model for atherosclerosis research. In this mini review, we summarize different zebrafish models used to study atherosclerosis, focusing on the latest applications of these models to the dynamic monitoring of atherosclerosis progression, mechanistic study of therapeutic intervention and drug screening, and assessment of the impacts of other risk factors.

## Introduction

Atherosclerosis (AS) is the pathological basis for many cardiovascular diseases (CVDs), presenting a severe threat to human health (Lusis, 2000; Thomas et al., 2018). AS is characterized by a large amount of lipid deposition in arterial blood vessels which forms plaques and causes muscular elastic artery stenosis, resulting in lumen occlusion or ruptured hemorrhage. The pathological process of AS is very complicated (Libby et al., 2011). First, excessive low-density lipoprotein (LDL) gradually accumulates underneath the endothelium and becomes oxidized LDL (Ox-LDL), which induces inflammation and results in excessive chemokines released from endothelial cells (Di Pietro et al., 2016). Attracted by chemokines, monocytes migrate to the intima of blood vessels and become macrophages upon the stimulation of macrophage colony-stimulating factors (M-CSFs) (Shao et al., 2016). Macrophages then engulf Ox-LDL and transform into foam cells, which release a number of factors that induce smooth muscle cell (SMC) migration and the formation of a fibrous cap (Sakakura et al., 2013). Subsequently, SMCs are gradually lost from the fibrous cap, while infiltrating macrophages degrade the collagen-rich cap matrix. These two mechanisms result in thinning of the fibrous cap, which leads to plaque rupture and thrombosis (Bentzon et al., 2014; Ziegler et al., 2019).

Animal models are essential for exploring the complex molecular mechanism of AS, which are conducive to studying the occurrence and development of AS, and to assessing the therapeutic effects of diet and drug interventions on AS (Fuster et al., 2012; Lee et al., 2017a). From the early 20th century to the era of new millennium, researchers have utilized a variety of animals in AS models for over 100 years, including rabbits, mice, rats, pigs and non-human primates (Lee et al., 2017a; Vedder et al., 2020). Large animal models such as pigs and non-human primates are suitable for AS research because their vascular lesion morphology and lipid metabolism are similar to that in humans (Rapacz et al., 1986; Getz and Reardon, 2012; Laila et al., 2018). The disadvantages of these models include long modeling times, high cost, complex experimental procedures, and difficulties in obtaining large amounts of data (Lee et al., 2017a). Small mammalian animals such as rabbits and mice are cheaper to rear, and mice can be easily manipulated genetically (Mushenkova et al., 2019; Poznyak et al., 2020). However, long-term fat-fed rabbits are prone to hepatotoxicity and a severe inflammatory response, and the plasma lipid profiles differ considerably among inbred strains of mice and also among different mouse mutants, rendering large variation in their susceptibility to AS (Paigen et al., 1985; Getz and Reardon, 2012; Fan et al., 2015; Golforoush et al., 2020; Vedder et al., 2020). Thus, no single animal model is sufficient for AS research; therefore, additional novel animal models are required.

## Zebrafish Models for Atherosclerosis Research

**FIGURE 1:**
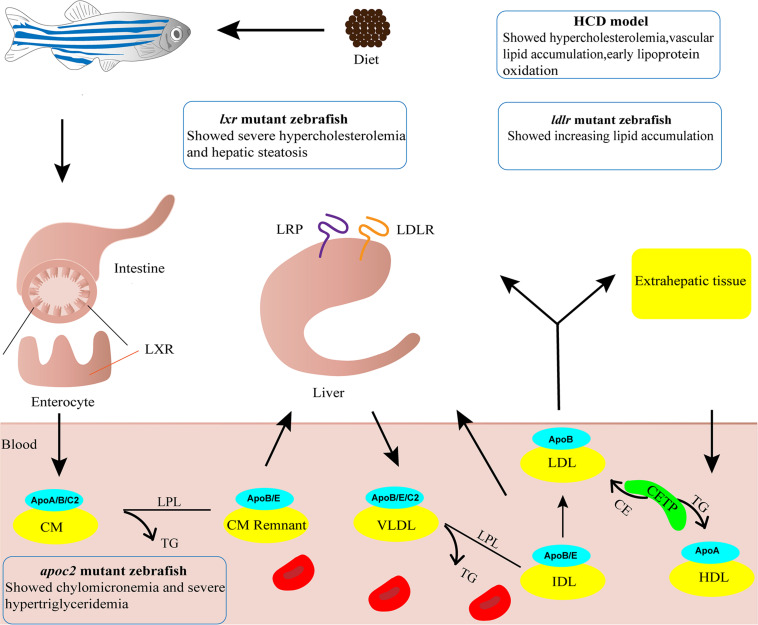
Zebrafish models for atherosclerosis research. Cartoon shows lipid metabolism and transport of lipoproteins in zebrafish. The phenotypes of four zebrafish models used in atherosclerosis research are indicated. ApoA/B/C2/E, Apolipoprotein A/B/C2/E; CE, cholesteryl ester; CETP, cholesteryl ester transfer protein; CM, chylomicron; CM Remnant, chylomicron remnant; HCD, high cholesterol diet; HDL, high-density lipoprotein; IDL, intermediate-density lipoprotein; LDL, low-density lipoprotein; LDLR, low-density lipoprotein receptor; LPL, lipoprotein lipase; LRP, low density lipoprotein receptor-related protein; LXR, liver x receptor; TG, triglyceride; VLDL, very-low-density lipoprotein.

Zebrafish have proven to be an excellent animal model for developmental studies and human disease modeling (Lieschke and Currie, 2007; Gut et al., 2017). The advantages of zebrafish, including small body size, low rearing cost, large numbers of offspring, *ex utero* fertilization and rapid development, render it feasible and affordable to perform large scale screening of candidate targets (Gut et al., 2017). Furthermore, embryonic transparency facilitates dynamic monitoring of various cellular processes *in vivo* through live imaging with fluorescent reporter lines (Lieschke and Currie, 2007; Holtzman et al., 2016). Zebrafish are poikilothermic vertebrates that favor lipids as a source of energy, whereas homeothermic mammals favor carbohydrates (Stoletov et al., 2009). Although the plasma lipid profiles differ largely between zebrafish and humans, the lipid metabolism process is highly conserved which has been extensively discussed in previous reviews (Holtta-Vuori et al., 2010; Fang et al., 2014; Schlegel, 2016; Figure 1). In short, the genes involved in lipoprotein and lipid metabolism, such as *apob*, *apoe*, *apoa1*, *ldlr*, *apoc2*, *lpl*, *lcat* and *cetp*, are conserved in zebrafish (Anderson et al., 2011; Fang et al., 2014). Notably, the Apob protein in zebrafish is equivalent to APOB100, not APOB48, in humans (Otis et al., 2015). Since LDL and very-low-density lipoprotein (VLDL) containing APOB100 have a longer half-life in plasma, zebrafish are more likely to develop AS (Babin and Vernier, 1989; Stoletov et al., 2009; Fang et al., 2010). Furthermore, cholesteryl ester transfer protein (CETP), an enzyme whose net effect favors AS development in humans, is expressed in zebrafish but not in mice (Fang et al., 2014). Compared to other AS animal models, the sharp increase in oxidized lipoproteins is most significant in zebrafish fed a high cholesterol diet (HCD), making them excellent models for investigating the mechanism of lipoprotein oxidation (Stoletov et al., 2009; Fang et al., 2010). However, zebrafish models also possess their own drawbacks. Blood samples can be collected only in small quantity from zebrafish that are older than 45 days, homogenates of several larvae are used instead for studies at early developmental stages. The lipoprotein profile and LDL makeup are different and no late-stage lesion is observed in zebrafish AS models (Stoletov et al., 2009; Fang and Miller, 2012, 2013). Nevertheless, zebrafish have become a popular alternative animal model for AS research, especially for early progression of AS (Holtta-Vuori et al., 2010; Vedder et al., 2020). Thus, several zebrafish models have been established and are widely used (Figure 1).

### High-Cholesterol Diet Model

Adult zebrafish subject to 8–12 weeks of 4% (w/w) HCD feeding are susceptible to developing hypercholesterolemia, with plasma total cholesterol level reaching 800 mg/dL, a 4-fold increase compared to normal diet (ND)-fed fish (Stoletov et al., 2009). Vascular lesions of enlarged intima with accumulation of lipid and cell infiltration can be found in sections of dorsal aorta. Such lesions are classified as fatty streaks which equal to type II AS lesions in humans, the early stage of type I-VI lesions (Stary et al., 1995; Stoletov et al., 2009). Upon a 10-day challenge with HCD, zebrafish larvae display vascular lipid accumulation, myeloid cell aggregation, and early lipoprotein oxidation (Stoletov et al., 2009; Fang et al., 2010). The vascular lipid accumulation is dose-dependent upon feeding various concentration of cholesterol (2–10%), with most reproducible results achieved at 4% cholesterol (Stoletov et al., 2009). Thus, the zebrafish HCD model is frequently used to study the early development of AS. However, a recent study reported that the 10-day challenge with HCD did not render accumulation of macrophages in the blood vessels of zebrafish larvae (Verwilligen et al., 2020). The reason for this discrepancy warrants further investigation but the difference suggests that longer HCD feeding as in adult fish may be necessary.

### *ldlr* Mutant Zebrafish

Deficiency in the LDL receptor (LDLR) results in familial hypercholesterolemia in humans (Andersen et al., 2017). Liu et al. knocked out *ldlr* in zebrafish using CRISPR/Cas9 technology. The *ldlr* mutant zebrafish with a ND feeding showed activation of SREBP-2 pathway and developed moderate hypercholesterolemia. After being fed a HCD for only 5 days, *ldlr* mutant larvae showed increased lipid accumulation in blood vessels and exacerbated hypercholesterolemia, which resulted in type II AS lesions (Liu et al., 2018; Vedder et al., 2020). The reduction in diet challenge time makes this model more useful for mechanistic studies and screening of potential drugs for hypercholesterolemia.

### *apoc2* Mutant Zebrafish

APOC2 is an activator of lipoprotein lipase that plays an important role in lipid metabolism (Wolska et al., 2017). Liu et al. (2015) knocked out *apoc2* in zebrafish using TALEN technology. The results revealed chylomicronemia and severe hypertriglyceridemia in *apoc2* mutants (Liu et al., 2015), which resembled the characteristics of patients lacking *APOC2* (Baggio et al., 1986). *Apoc2^–/–^* larvae fed a ND showed lipid accumulation and lipid-containing macrophages in the vasculature, similar to that observed in the development of human type II AS lesions (Liu et al., 2015; Vedder et al., 2020). As a result, *apoc2^–/–^* zebrafish without HCD feeding can serve as dyslipidemia models to investigate the molecular mechanism of LDL infiltration, oxidation and phagocytosis by vascular wall macrophages, and used as models for screening of potential drugs for hyperlipidemia.

### *lxr* Mutant Zebrafish

Liver X receptors (LXRs) plays important roles in cholesterol catabolism (Calkin and Tontonoz, 2012). There are two subtypes of LXRs in mammals, LXRα and LXRβ, and only one *lxr* gene with higher homology to LXRα is present in zebrafish (Pinto et al., 2016). Zebrafish with a targeted deletion in *lxr* showed a substantial increase in LDL when fed a HCD or a high-fat diet (HFD), developing severe hypercholesterolemia and hepatic steatosis, with lipid deposition resembling fatty streaks in humans (Cruz-Garcia and Schlegel, 2014; Vedder et al., 2020). This model can be applied for screening intestinal-restricted LXR agonists, which can be used to suppress the development of dyslipidemia and atherosclerosis.

## Dynamic Monitoring of as Progression

There are several theories of AS pathogenesis, including endothelial injury, smooth muscle cell migration and proliferation, and monocyte-derived foam cell formation (Lusis, 2000; Libby et al., 2013; Sakakura et al., 2013; Emini Veseli et al., 2017). AS is also related to oxidative stress, non-coding RNA, inflammation (Feinberg and Moore, 2016; Kattoor et al., 2017). However, no single theory clearly elaborates the progression of AS (Libby et al., 2019). It is of great importance to explore the complex and diverse pathophysiological processes of AS, especially the spatiotemporal specificity. Mammalian models, such as mice and rabbits, and human biopsy samples, can provide only endpoint results (Lee et al., 2017a,b), while zebrafish can be used to monitor the development of AS dynamically using *in vivo* live imaging because of their embryonic transparence and an abundance of fluorescent reporter lines (Fang and Miller, 2012; Gut et al., 2017).

Luo et al. (2019) fed *Tg(lysc:EGFP)*, *Tg(mpx:EGFP)*, and *Tg(mpeg1:EGFP)* transgenic zebrafish lines HCD to dynamically monitor myeloid cells/neutrophils *in vivo* during the initial stage of atherosclerosis. They also enriched endothelial cells with green fluorescence from HCD fed *Tg(flk1:EGFP)* fish by flow cytometry to detect gene expression (Luo et al., 2019). The results showed that endothelial cell inflammation occurred as an early pathological change, which was prior to the accumulation of myeloid cells and neutrophils in blood vessels. Lipid depositions occurred after neutrophil accumulation. Pharmacological inhibition of HCD-induced early endothelial inflammation may prevent the initial development of AS (Luo et al., 2019).

Additionally, the HCD model is used to explore the role of Ox-LDL during the early stage of AS. Oxidation events, such as malondialdehyde (MDA) formation, produce immunogenic epitopes, and specific antibodies have been used in the study of AS and cardiovascular imaging (Miller et al., 2011). Fang et al. (2011) generated a transgenic zebrafish line with a heat-shock-promoter-driven EGFP labeled single-chain monoclonal antibody, IK17, which can bind to MDA-LDL. *In vivo* imaging demonstrated that continuous expression of IK17-EGFP decreased HCD-induced lipid accumulation in the blood vessel wall, suggesting antioxidant antibodies may exert a therapeutic benefit. This model also provides an effective method for testing the therapeutic outcome of diets and other oxidation-specific antibodies, which may eventually be applied to human (Fang et al., 2011).

Recently, Thierer et al. (2019) developed a reporting system, LipoGlo, to monitor atherosclerotic lipoproteins sensitively and specifically. The system used a luciferase enzyme (NanoLuc) fused with ApoB to monitor the abundance, size and location of lipoprotein particles, which are decisive factors in AS (Thierer et al., 2019). The authors described the lipoprotein profiles of individual zebrafish larvae, collected images of atherosclerotic lipoprotein localization and reported multiple extravascular lipoprotein localization modes. Finally, through this system, Pla2g12b was determined to be an effective regulator of lipoprotein size (Thierer et al., 2019).

## Mechanistic Study of Therapeutic Intervention and Drug Screening

Studies have shown that apolipoprotein plays important roles in the prevention and treatment of AS (Peng and Li, 2018). The overexpression of apolipoprotein A-I binding protein (AIBP) mitigates diet-induced metabolic abnormalities, reducing diet-induced lipid accumulation in zebrafish blood vessels, and showing a protective effect from AS (Schneider et al., 2018). In addition, ezetimibe promotes the expression of apolipoprotein A-II through the HNF4 and PPARα transcription factor in the HCD model (Yan et al., 2019). However, ezetimibe did not inhibit vascular lipid accumulation or macrophage recruitment induced by HCD when apolipoprotein A-II was knocked out, implying that apolipoprotein A-II plays a pivotal role in reducing AS caused by HCD (Yan et al., 2019). The effects of several drugs, including atorvastatin, fenofibrate and ezetimibe, on the cholesterol levels in HCD-fed zebrafish model have been investigated (Chen et al., 2017). Intravascular cholesterol levels were significantly increased after HCD feeding and decreased after drug treatment. Atorvastatin exerted effects in a concentration-dependent manner, while only intermediate and highest concentration of fenofibrate and ezetimibe had effects in this model (Chen et al., 2017). Another study showed combination of low doses of ezetimibe and simvastatin may have an additive effect in reducing cholesterol levels in zebrafish (Baek et al., 2012).

Drug screening is important for the discovery of novel therapeutic interventions for AS (Foks and Bot, 2017). Recent studies have identified many substances, for examples, water extracts of cinnamon, turmeric, laurel, clove, grape skin, lophatherum herb, and acai berry puree, as well as the traditional Chinese medicine monomer chrysophanol, all of which show anti-atherosclerotic activity in zebrafish models (Jin and Cho, 2011; Jin et al., 2011; Kim et al., 2012). The latest research revealed that indole-3-methanol (I3C), a cruciferous vegetable extract, inhibits lipid deposition in hyperlipidemic zebrafish larvae by activating autophagy (Jiang et al., 2019). In addition, solid-state fermented polysaccharides from king oyster mushroom had an apparent positive influence on the inhibition of the Ox-LDL induced development of foam cells from mouse macrophages and exerted a lipid-reducing effect in the lipid absorption stage of a zebrafish hyperlipidemia model (Wei et al., 2018).

## Assessment of as Risk Factors

AS is a progressive chronic inflammatory disease. It is not only affected by pathogenic factors, such as diabetes, chronic kidney disease, hypertension and hyperlipidemia, but is also related to other risk factors, such as genetics, age, gender, smoking, obesity, diet and nutritional status (Hughes et al., 2013; Herrington et al., 2016). As a focus of therapeutic intervention in the future, it is of great importance to assess the effects of environmental factors, trace elements, drugs and other risk factors on AS pathogenesis.

### Environmental Factors

Environmental pollution is becoming more serious worldwide, and soil cadmium pollution is one such problem (Genchi et al., 2020). Cadmium absorbed by soil can be ingested by humans through the food chain (Jaishankar et al., 2014). As a heavy metal, cadmium is harmful to human health (Edwards and Prozialeck, 2009; Lin et al., 2016), but its specific effect on lipoproteins is unknown. Researchers fed zebrafish a HCD containing cadmium chloride, and discovered that exposure to cadmium influenced the function of high-density lipoprotein (HDL), which further resulted in hyperlipidemia, inflammation, fatty liver changes and increased CETP activity in HCD zebrafish (Kim et al., 2018).

According to WHO statistics, outdoor air pollution, such as fine particulate matter with a diameter ≤2.5 μm (PM2.5), causes approximately 3.7 million deaths every year. Long-term exposure to high concentrations of PM2.5 increases the risk of cardiovascular diseases and cancers (Kloog et al., 2013; Dabass et al., 2016; Turner et al., 2020). PM2.5 has been previously reported to cause disorders in zebrafish lipid metabolism (Kim et al., 2015). Duan et al. used transcriptomics to analyze the differential expression of mRNA and microRNA. The results revealed that PM2.5 upregulates let-7b, miR-153b-3p, miR-122 and miR-24, while downregulating let-7i, miR-19a-3p, miR-19b-3p and miR-7a, indicating that PM2.5 may activate autophagy, impair vasodilation, and cause cardiovascular-related diseases, such as hypertension, atherosclerosis, and myocardial infarction (Duan et al., 2017).

### Trace Elements

Trace elements play indispensable roles in metabolism. Iron is involved in various physiological processes, such as red blood cell function, oxygen transport, DNA synthesis, protein synthesis, and cell replication. Iron deficiency can lead to anemia, neurodegenerative disease, developmental delay and behavioral disorders, while excessive iron may also adversely affect health (Tsay et al., 2010; Wang and Pantopoulos, 2011; Radlowski and Johnson, 2013). Lipoprotein and iron interact with each other in the form of ferrous or ferric ions in blood (Kim et al., 2017). LDL and HDL are easily oxidized and modified by ferrous ions to produce additional atherosclerosis-related proteins (Kim et al., 2017). Studies showed that after 24 weeks of iron consumption, both ND-fed and HCD-fed zebrafish displayed significant increases in serum cholesterol and triglyceride levels, which for the first time demonstrated the influence of iron consumption on lipid homeostasis in a zebrafish model (Kim et al., 2017). It was reported that inhibition of ferroptosis alleviated AS in ApoE*^–/–^* mice, through attenuating lipid peroxidation and endothelial dysfunction in aortic endothelial cell (Bai et al., 2020). However, there is no report on ferroptosis in zebrafish AS models yet.

### Drugs

Quinolones and tetracyclines are β-diketone antibiotics that are widely used to treat infectious diseases in human and animals (Aminov, 2017). Wang et al. (2018) treated zebrafish with β-diketone antibiotics and discovered that upregulation of miR-125b and miR-144 led to dyslipidemia that caused increased cholesterol content, thereby elevating the risk of zebrafish developing hyperlipidemia and atherosclerosis.

### Other Risk Factors

Artificial sweeteners have been widely consumed, but problems with their safety have not been fully addressed, nor have their effects on AS progression been delineated (Gardener and Elkind, 2019). Recently, researchers revealed that zebrafish fed HCD and aspartame exhibited acute swimming deficiency and brain inflammation. Furthermore, the serum lipid profile changed with increasing CETP levels in a zebrafish AS model fed saccharin (Kim et al., 2011).

## Prospects

AS is a complex and progressive disease for which the pathogenesis is not fully understood. Current clinical remedies for AS include statins, antithrombotic drugs and surgical interventions to alleviate the complications, but there are still no effective or specific drugs for AS treatment (Fukuda et al., 2015; Kobiyama and Ley, 2018). Zebrafish models may aid in understanding AS pathogenesis and the identification of novel therapeutic targets and approaches (Kobiyama and Ley, 2018). Current established zebrafish models for AS research are generally fed a short-term HCD and/or HFD, resulting in models corresponding to the early stage of AS. Furthermore, most studies utilize the optical transparency of embryonic and larval zebrafish for *in vivo* live imaging, which has neglected the use of adult fish model (Gut et al., 2017). Therefore, more attention should be paid in the future to generating models that can mimic middle and late stages of AS and building adult fish models, for example combination of HCD with other diets such as vitamin C-deficient diet which leads to robust oxidative stress and accelerated atherosclerotic process (Kirkwood et al., 2012), or generation of transgenic and knockout animals which render the plaques vulnerable to rupture. This will ensure the screening of new drugs or therapeutic targets in zebrafish more relevant for human AS patients.

## Author Contributions

DT and FG reviewed the literature. DT, FG, CY, and RZ wrote and revised the manuscript. All authors contributed to the article and approved the submitted version.

## Conflict of Interest

The authors declare that the research was conducted in the absence of any commercial or financial relationships that could be construed as a potential conflict of interest.

## Abbreviations

AIBP: A-I binding protein
AS: atherosclerosis
CETP: cholesteryl ester transfer protein
CVDs: cardiovascular diseases
HCD: high cholesterol diet
HDL: high-density lipoprotein
HFD: high fat diet
I3C: indole-3-methanol
LDL: low-density lipoprotein
LXRs: liver X receptors
MDA: malondialdehyde
M-CSF: macrophage colony-stimulating factors
ND: normal diet
Ox-LDL: oxidized LDL
VLDL: very-low-density lipoprotein.
